# Singularity Containers Improve Reproducibility and Ease of Use in Computational Image Analysis Workflows

**DOI:** 10.3389/fbinf.2021.757291

**Published:** 2022-01-27

**Authors:** Shilpita Mitra-Behura, Reto Paul Fiolka, Stephan Daetwyler

**Affiliations:** ^1^ Lyda Hill Department of Bioinformatics, UT Southwestern Medical Center, Dallas, TX, United States; ^2^ Department of Cell Biology, UT Southwestern Medical Center, Dallas, TX, United States

**Keywords:** singularity container, reproducibility, imaging facilities, software dissemination, code sharing, ease of use, high-performance cluster computing

## Abstract

Reproducing computational workflows in image analysis and microscopy can be a daunting task due to different software versions and dependencies. This is especially true for users with little specific knowledge of scientific computation. To overcome these challenges, we introduce Singularity containers as a useful tool to run and share image analysis workflows among many users, even years later after establishing them. Unfortunately, containers are rarely used so far in the image analysis field. To address this lack of use, we provide a detailed step-by-step protocol to package a state-of-the-art segmentation algorithm into a container on a local Windows machine to run the container on a high-performance cluster computer.

## Introduction

The need for computational image analysis has drastically increased over the past decade. Modern microscopes are now able to simultaneously observe multiple samples such as zebrafish or mouse embryos over several days at single cell resolution (McDole et al., 2018; Daetwyler et al., 2019). This leads to complex and large datasets that are often tens to hundreds of Terabytes in size. Consequently, manual inspections and analysis of the acquired data are less and less feasible. Instead, automated image processing and analysis workflows are becoming indispensable for handling the acquired data.

To deal with these complex and large datasets, image processing and analysis workflows are often sophisticated and consist of multiple steps. In addition, they often require powerful computational resources such as high-performance cluster computation (Daetwyler et al., 2019). This poses a considerable challenge to scientists with no previous background in scientific computation. This is particularly an issue in imaging facilities where many users with various backgrounds have access to state-of-the-art microscopes. Ideally, such facilities would provide users with robust, yet simple interfaces with no need to install or set up software to enable complex and thorough workflows dedicated to specific microscopes.

Additionally, software versions and their dependencies change fast. By the time a researcher wants to reproduce an established pipeline for their own dataset, e.g., from a published research paper, the required software or its dependencies may have changed. Therefore, reproducing the results may be time-consuming at best or impossible at worst. For example, if a reader is trying to recreate an experiment that was published in 2013 with code written in Python 2.7.6 but has Python 3.8 installed locally, they might have to revert to 2.7.6 or find another workaround to properly run the code.

To overcome these issues, one convenient workaround is to use containers. Containers are packaging mechanisms for software that allow a workflow to be separated from the original system it was created and run on. Therefore, containers can immortalize the specific software state needed to run an original app or pipeline. This is ideal for later reproducibility and code sharing. From a user perspective, this means that instead of taking and modifying many lines of code, correctly downloading and setting dependencies, and dealing with future updates of utilized software packages, only downloading the container image and a few lines of code are required to reproduce a pipeline.

Despite all these advantages, containers are rarely used in the imaging field, as building a container may appear difficult at first glance. To support the bioimaging community, we describe here the advantages of containers in detail and how to establish a container for an image analysis pipeline with a step-by-step protocol. Ultimately, we hope that this methods paper will increase data sharing and reproducibility with the help of containers in advanced workflows in microscopy labs and imaging facilities.

## Singularity Containers are Well Suited for High-Performance Computing

To understand the concept of a container, it is important to understand the building blocks of a Unix operating system (Linux Information Project 2005). User interactions such as writing in a text editor or image processing software run in the so-called user-space. In a distinct and protected part of the system memory, the kernel (i.e., the core of the operating system) controls device drivers and executes processes such as Input/Output (I/O), e.g., data transfer to and from a CPU, or to and from peripheral devices such as a keyboard. Containers utilize the machine’s operating system kernel and only segregate processes in user-space (Turnbull 2014; Young 2017). This enables the coder to have multiple isolated user-space instances on one single machine in a “lightweight” way that avoids the overhead of virtual machines (VMs), which are an abstraction of the physical hardware and include a full copy of the operating system with its own kernel.

One of the most widely used container virtualization tools is called Docker, with a community of over 11 million developers (Docker, Inc. 2021a). Docker is very robust in its ability to create and run containers and is easily used on local systems and shared with other users through their online hub (https://hub.docker.com/). However, Docker containers, as well as most other container solutions, traditionally require root privileges to be built and run. This presents an issue on High-Performance Computing (HPC) clusters required for advanced image analysis workflows as most users do not have root access. Although Docker now offers a rootless mode, several limitations currently exist for the rootless mode in Docker, including a limited amount of supported storage drivers (Docker, Inc. 2021b). To offer a suitable alternative for HPC clusters, Singularity containers were developed (Kurtzer, Sochat, and Bauer 2017). Importantly, Singularity containers do not need root access to the host system to run and are therefore widely adopted on HPC clusters. Moreover, to interact with the huge Docker community and pre-built containers, Singularity is able to build most Docker images pulled from Docker Hub. In conclusion, Singularity is a great tool to package complex image analysis workflows into one container for HPC clusters.

## Workflow for Image Analysis Using Containers

**FIGURE 1:**
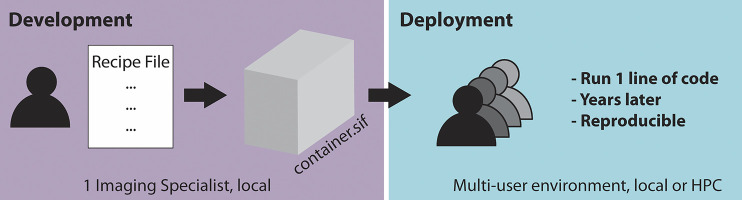
Schematic of a two-step workflow for the usage of containers: “build once, apply forever.”

Singularity containers are particularly useful if many users in a microscopy lab or imaging facility require the same workflow repeatedly. This is often the case as the same microscopes are used for acquisitions, requiring the same workflow for image processing and further analysis tasks. In an ideal scenario (Figure 1), one dedicated imaging specialist, who is familiar with programming, develops a container containing the required processing workflow for one or several microscopes. The established container image is then shared with the many end-users of the microscope or other users outside of the facility. The end-users then only need a few lines of code to start running the container. This enables the end-user to not have to deal with dependencies or similar issues to be confident that the appropriate workflow is applied to the acquired data. Moreover, by saving the container, the applied analysis can be repeated by other users years later, which is expected to significantly improve reproducibility in image processing.

To exemplify the usefulness of a container, we have containerized a workflow that includes a recently published segmentation tool, Cellpose (Stringer et al., 2021). Cellpose has shown great promise across many biological applications and might therefore be a valuable tool for many microscopists. In our exemplary container, we have built a container that consists of a workflow of first segmenting images from a selected folder, and saving them into a new, user-defined folder in a second step. To set the parameters for a successful segmentation, our container can directly receive inputs from the command line after the run container call (Code 18).

## Material and Equipment

An end user in an imaging facility only requires a container and a Linux operating system (e.g., on the HPC cluster) with Singularity installed to run it (Code 1, Code 18). Singularity is likely already available on your HPC cluster. Alternatively, contact the HPC administrators or follow the description on https://sylabs.io/guides/3.7/admin-guide/installation.html# to install Singularity. With Singularity installed, any user can directly run a compiled Singularity Image File (SIF) indicated with a “.sif” file extension with (Code 1):


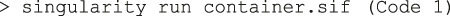


To establish a container, an image analysis specialist is required. To build a Singularity container locally (c.f. Supplementary Note S1 for building a container on the cluster), you either need to have a Linux system or create a Linux virtual machine. As most microscopy labs and imaging facilities rely on Windows on their local workstations, we provide a step-by-step protocol to set up a virtual machine and build a Singularity container for the use on a HPC cluster on Windows below. For this, the following software is needed:

1) Install git with default settings: https://gitforwindows.org/(for the Git Bash), which provides a terminal to build the container.
2) Install a VirtualBox with default settings (https://www.virtualbox.org/wiki/Downloads), which provides a virtual machine in which Linux will run to build the container.
3) Install Vagrant (https://www.vagrantup.com/downloads.html), which automates the VM setup and makes a Virtual-Box with pre-installed Singularity available for use.
4) (Optionally) Install Vagrant Manager (https://www.vagrantmanager.com/downloads/), which centralizes the VMs for easier handling, particularly when building several containers in parallel.

## Step-by-Step Protocol

**FIGURE 2:**
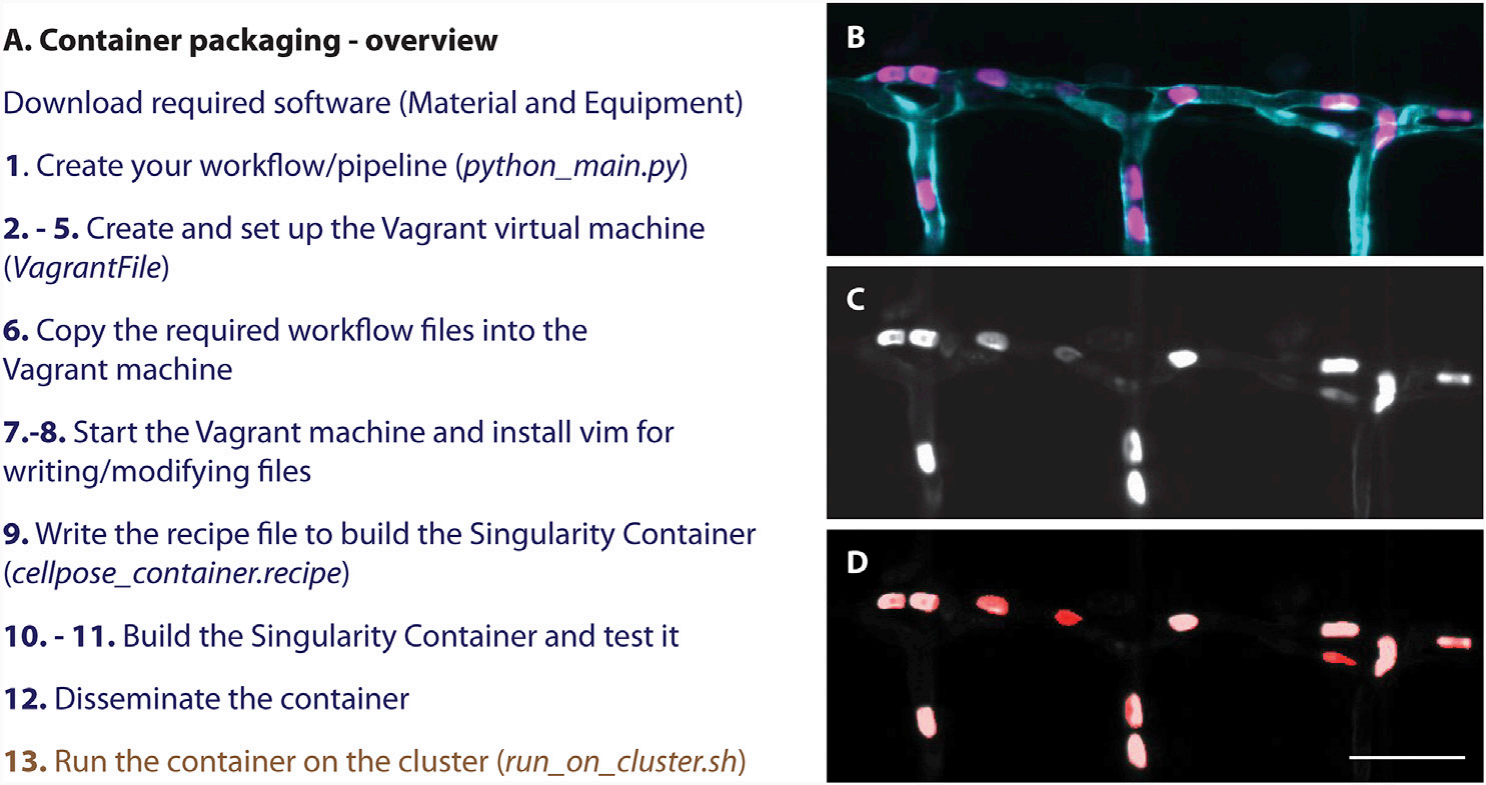
**(A)** Overview of a step-by-step protocol to establish a Singularity container. While an image analysis specialist of a facility is required to follow all steps (blue), an end user only needs to run the container (brown) and therefore does not have to deal with setting up the software and its dependencies correctly. **(B–D)** Resulting segmentation from applying the here described container: **(B)** Still image of red blood cells (magenta) labelled with Tg (Gata1a:dsRed) (Traver et al., 2003) in a zebrafish embryo with a vascular marker (cyan) Tg (kdrl:eGFP) (Jin et al., 2005). **(C)** Grey scale image of the labelled red blood cells **(D)** Resulting segmentation (red) overlayed on the grey scale image **(C)**. Scalebar: 50 um.

To successfully build the above described container containing our workflow based on Cellpose (Stringer et al., 2021) (1), we provide a step-by-step protocol here (Figure 2, Table 1). For this, first, a virtual environment on Windows has to be set up containing all required files (2–8), before building the container (9–11) and disseminating and applying it (12–13, Figure 2B). All files to successfully build the container are available as supplementary or on github: https://github.com/DaetwylerStephan/Containerize_ImageAnalysis/.

1) Create/design your workflow for image analysis. For demonstration purposes, we wrote a Python file “python_main.py” (c.f. Supplementary Files, github) that contains the relevant commands for a simple segmentation workflow using Cellpose (Stringer et al., 2021), a state-of-the art segmentation algorithm.
2) Next, open Git Bash and go to or make the directory (Code 2) in which you want to build your container. The directory that is being used for this demonstration is called “container_example.” Copy the python_main.py file into the container_example folder.

**TABLE 1:** Code used in the step-by-step protocol for establishing a container using a definition file. Please note that copying some code directly from the table might induce errors due to formatting. Hence, we suggest to type the commands newly in case of errors, and refer to the files on github: https://github.com/DaetwylerStephan/Containerize_ImageAnalysis or in the Supplementary Material.

Code	Project title
Code 2	> mkdir container_example/
	> cd container_example/
Code 3	> vagrant init sylabs/singularity-3.7-ubuntu-bionic64
Code 4	> vim Vagrantfile
Code 5a	config.vm.synced_folder "C:/your/local/cellimages/filepath", "/home/vagrant/cellpose_testdata"
Code 5b	config.vm.provider "virtualbox" do |vb| #Customize the amount of memory on the VM: vb.memory = "9000" end
Code 6	> vagrant up
Code 7	> vagrant ssh-config
Code 8	> scp -P 2222 python_main.py vagrant@127.0.0.1:.
Code 9	> vagrant ssh
Code 10	vagrant@vagrant> sudo apt-get update
	vagrant@vagrant> sudo apt-get install vimvagrant@vagrant> vim cellpose_container.recipe
Code 11	Bootstrap: docker From: ubuntu:18.04
Code 12	%labels AUTHOR: Shilpita Mitra-Behura, Reto Fiolka, Stephan Daetwyler
Code 13	%files /home/vagrant/python_main.py /mnt
Code 14	%post #Downloads the latest package lists. apt-get update -y
	#Install python and other tools. #Non-interactive is used to ensure prompts are not needed. DEBIAN_FRONTEND=noninteractive apt-get install -y --no-install-recommends \ python3 python3-pip\ python3-setuptools
	#Update pip python3 -m pip install --upgrade pip==21.2.4
	#Install python libraries needed for Cellpose to run python3 -m pip install wheel==0.37 python3 -m pip install --no-cache-dir torch==1.8.1 python3 -m pip install opencv-python-headless==4.5.3.56 python3 -m pip install cellpose==0.6.5
Code 15	%runscript
	exec /usr/bin/python3 /mnt/python_main.py “$@”
Code 16	%help This is a Singularity container to segment images using Cellpose. To run this container, use, for example, 'singularity run cellpose_container.sif --filedir /folder/where/files/to/segment/are --savedir /folder/where/segmented/files/should/be/saved --pretrained_model cyto --chan 2 --save_tif'
	The user must put the folder where the files to be segmented are after the flag “--filedir.” The directory where the user wants to save their files is optional, with the tag “--savedir.” The flag “--pretrained_model” is required and must have either the input “cyto” or “nuclei.” The flags “--chan” and “--chan2” are optional and allude to the channels of the image that will be segmented. The flag “--diameter” is optional and can be added for the user to specify the diameter of the nuclei in image. The flags “--flow_threshold”, “--cellprob_threshold” are further optional flags to specify cellpose parameters. The flags “--save_png” and “--save_tif” are used to denote what file type the segmented files are saved as. The default is to save as a Tiff.
Code 17	vagrant@vagrant> sudo singularity build cellpose_container.sif cellpose_container.recipe
Code 18	vagrant@vagrant> singularity run cellpose_container.sif --filedir /home/vagrant/cellpose_testdata --savedir /home/vagrant/cellpose_testdata/save_segmentedimages --pretrained_model nuclei --chan 1 --flow_threshold 0 --cellprob_threshold −1 --diameter 19 --save_tif
Code 19	> scp -P 2222 vagrant@127.0.0.1:/home/vagrant/cellpose_container.sif .
Code 20	> sbatch run_on_cluster.sh


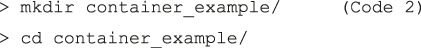

3) After creating this working folder, initialize a Vagrant virtual machine established for Singularity in Git Bash (Code 3). This sets up a Linux based virtual machine with Singularity already installed. To confirm that a Vagrant virtual machine has been set up successfully, check whether a “Vagrantfile” was placed in the working directory by typing “ls”.

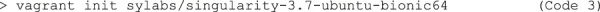

4) To establish a container that runs Cellpose on a set of images, mount a folder with test images directly to Vagrant for easier troubleshooting. To do so, use vim to open the Vagrantfile for editing (Code 4).

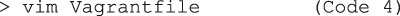


In the Vagrant file, un-comment the “config.vm.synced_folder” line and add the path to your test images. Thereby, the first argument of “config.vm.synced_folder” is the path to the test images on the host system and the second argument is the path in the Vagrant/guest system (Code 5a). If the path you designate in the Vagrant system does not exist, it will be created for you. Note: If you are using Windows and copy your file path directly from your system, you must change backward slashes to forward slashes (as shown in Code 5a), or your Vagrant system will not compile.


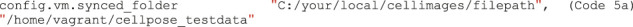


Additionally, modify the Vagrantfile memory setting “vb.memory” to ensure that there is enough RAM, by uncommenting the lines ‘config.vm.provider “virtualbox” do |vb|’, ‘vb.memory=”1024”’, and “end”. Cellpose requires at least 8 GB of memory allocation, so provide at least that much memory (Code 5b). A screenshot of a modified Vagrantfile can be found in the Supplementary Note S2.


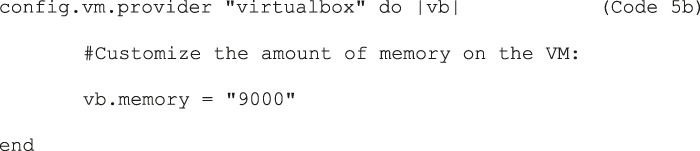

5) After saving this Vagrantfile, run “vagrant up” to build the virtual machine (Code 6). If your workstation is behind a corporate firewall, proxy issues might occur. In that case, download the vagrant box directly (Supplementary Note S3A).

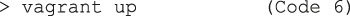

6) The Python file must be moved into the Vagrant system so that Singularity can access it when building the container. To copy the Python file into Vagrant, obtain the HostName and port of the Vagrant machine (Code 7).

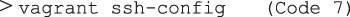


Next, securely copy (scp) the Python file into Vagrant (Code 8),in our case with HostName:127.0.0.1, port: 2,222. The password for Vagrant is “vagrant”.


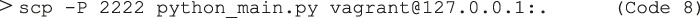

7) Now run “vagrant ssh” to connect to the virtual machine (Code 9). Once the virtual machine has started, check that the folder with test images has been bound properly and that the python_main file is available. If you enter the command “ls”, “python_main.py” and the folder “cellpose_testdata” with your test images should be listed.

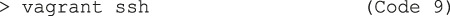

8) Next, install vim (Code 10) in the Vagrant environment to be able to write the definition file (also known as build recipe) to build a Singularity container. Make sure to enter “Y” when prompted by the terminal.

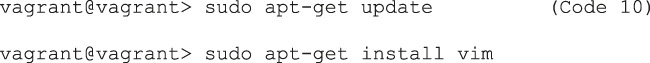

9)Now use vim to write the definition file “cellpose_container.recipe” for building the container, or copy a definition file into Vagrant the same way as the “python_main.py” file. If you copy the example definition file provided into your vagrant box, exit the vagrant box first by typing “exit”. The complete definition file for our Cellpose container is available in the Supplementary Files or on github. In our example, the definition file was composed of the following sections:
9a)The Singularity definition file contains a bootstrap specification header (Code 11). This header indicates which Singularity module is used to set up the core components of the container (Kurtzer, Sochat, and Bauer 2017). Thereby, we can benefit from already established images, and hence use an established Docker image as an underlying template which contains important container settings. In addition, we specify a version of the Unix operating system which is run in the container.

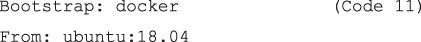

9b)Next, write labels (%labels) in the definition file to annotate your container. For example, specify the author names. (Code 12)

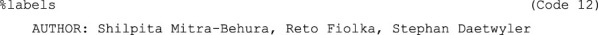

9c)Next, write the %files section in the container to specify which files have to be copied into the container. We specified that the python_main.py file, saved in the Vagrant Box, is moved into the /mnt folder of the container so that it can be called from within the container. (Code 13)

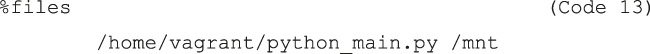

9d)Next, write the %post section (Code 14). This section is executed once while the container is built and run from inside the container. This means that all required software to run the imaging pipeline has be installed here. As Cellpose is a Python library, provide the commands to install Python, all required Python dependencies of Cellpose, and Cellpose itself here. It is good practice to provide version numbers, e.g., cellpose==0.6.5, for later reproducibility.

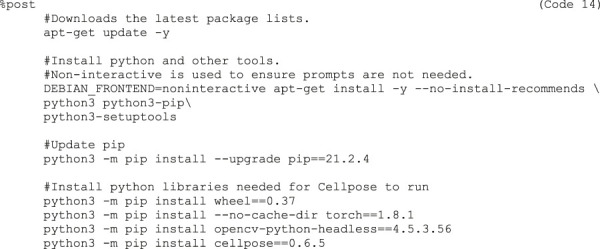

9e)Next, write the runscript (Code 15). This is the part that is run when the container is executed by the user.

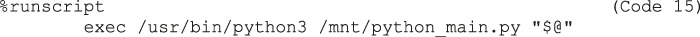

9f)Next, provide a help section (Code 16). This section is important for users to understand how to use the container and can be called by entering “singularity run-help cellpose_container.sif”:

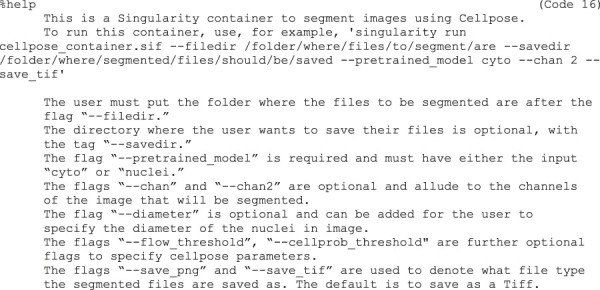

9g)Save the file and exit vim.
10)With the complete definition file “cellpose_container.recipe”, it is time to build the container (Code 17), where the first argument is the name of the Singularity image that is built and the second argument is the container definition file. Building the container, especially in a Vagrant system with limited RAM, is time-consuming and can take several minutes. In case of proxy issues (e.g., “conveyor failed to get”—error message), the Vagrantfile should be modified before building the container (Supplementary Note S3B).

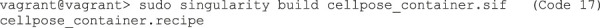

11) Test the newly built container on the images mounted to the Vagrant directory (Code 18). When running this code, Cellpose will download the available models first before segmenting. Pay attention to the Cellpose documentation to ensure that the arguments you supply to the container make sense for your data (i.e., pay attention to which channels are used in your input images). Note: Currently, the workflow does not overwrite older files, so make sure to delete older files if you are running the code on the same files and saving into the same directory twice. *Note 2*: Copying Code 18 from Table 1 to the command line might introduce errors due to the hyphens. Therefore, we suggest typing it newly.

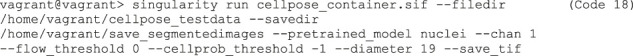

12) Next, type “exit” in the vagrant prompt to exit the Vagrant virtual machine. Using Code 7, find the port information (e.g. HostName:127.0.0.1, port: 2,222) to copy the Singularity container file to your local host computer folder “.” (Code 19). The password is “vagrant”. From there copy the container to your HPC or distribute it.

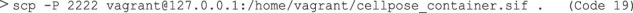

13) Now, apply the Singularity container containing Cellpose on available images on a high-performance cluster computer (Figure 2). For this, you need a SLURM file to submit a job to the cluster. This file should contain all required SLURM parameters, including parameters for job name, number of nodes, memory requirements and time limit. In addition, the SLURM file should load Singularity and run the container on selected images (c.f. Supplementary Files, github). Copy the SLURM file and the container to a folder on the cluster and go to this folder on the cluster. Now, run the container by calling the SLURM file (Code 20).

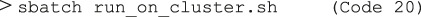


## Sandbox Container as Tool to Trouble-Shoot Container Development

Using definition files to build Singularity containers can be limiting. To check the functionality of your container, you must rebuild the container after every edit of the definition file. To address this issue, Singularity offers a convenient mode to build your container interactively and iteratively. This mode is known as sandbox mode. It creates a container within a writable directory. The directory thereby will act as a Singularity image file, and is mutable, unlike normal Singularity images (i.e., sif files). After you are satisfied with the setup of your directory (that is, your container), you can build a .sif file, which is immutable, for ease of use. Below we will briefly illustrate how to build and edit this writable directory to fulfill the same functions as the container we built in the previous section (Table 2).

A. First, follow steps 1–8 from above to establish a virtual Linux environment with Singularity on Windows. Please note that installing vim as part of step 8 is not necessary here.
B. Next, instead of writing a definition file, generate a writable directory, the sandbox container (Code 21). Here, we initialize an Ubuntu version 18.04 as basis for the sandbox container. Be aware that this code will overwrite other directories with the same name.

**TABLE 2:** Code used for establishing a sandbox container. Please note that copying text from the table might introduce some errors, particularly for the hyphens. In case of errors, we suggest typing the code newly in the command line.

Code	Project title
Code 21	vagrant@vagrant>sudo singularity build --sandbox nameofdir/ docker://ubuntu:18.04
Code 22	vagrant@vagrant> sudo singularity exec --writable nameofdir/ /bin/bash
Code 23 (alternatively)	vagrant@vagrant> sudo singularity shell --writable nameofdir/
Code 24	Singularity>
Code 25	Singularity> apt-get update -y
Code 26	Singularity> apt-get install -y python3
Code 27	Singularity> apt-get install python3-pip
Code 28	Singularity> python3 -m pip install --upgrade pip==21.2.4
Code 29	Singularity> pip install cellpose==0.6.5
Code 30	vagrant@vagrant> sudo cp python_main.py nameofdir/mnt/
Code 31	vagrant@vagrant> singularity exec nameofdir/ python3 /mnt/python_main.py --filedir /home/vagrant/cellpose_testdata --pretrained_model nuclei --chan 1 --save_tif
Code 32	vagrant@vagrant> sudo singularity build containername.sif nameofdir/
Code 33	vagrant@vagrant> singularity exec containername.sif python3 /mnt/python_main.py --filedir /home/vagrant/cellpose_testdata --savedir /home/vagrant/cellposedata/masks --pretrained_model nuclei --chan 1 --save_tif


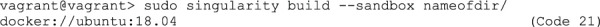

C. After building this sandbox container, shell into the directory to edit it. You can use either Code 22 or Code 23. The prompt should look like Code 24 after shelling into the directory.

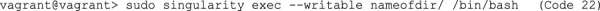



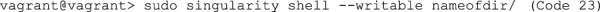



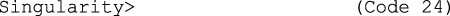

D. Now, update the repository package to get the information on the newest versions of packages and their dependencies (Code 25).

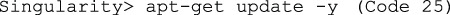

E. Next, we need a functional Python version. In our definition file above, we used the Python version 3.6.9 that comes with Ubuntu18.04 by default. Also, for the sandbox, install the default Python 3.6.9 (Code 26). Test whether it has been successfully installed with typing “python3 --version”.

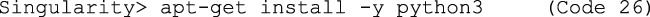

F. Then, install pip (Code 27) to install Python packages, and update pip (Code 28). The importance of updating pip is nicely explained in this blog post: https://pythonspeed.com/articles/upgrade-pip. In short, code builders can generate a compiled version of their code (“wheels”) and provide it for download in the Python Package Index. The pip in Ubuntu 18.04 is too old to recognize new wheel variants and thus will yield errors.

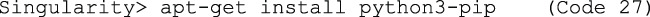
 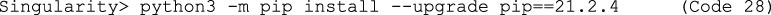


G. Use the upgraded pip to install Cellpose (Code 29).


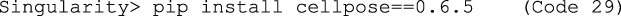

H. Exit the shell by typing “exit.” Copy python_main.py into the writable directory (Code 30). You can put it into whichever directory you like aside from root—we put ours in “/mnt.”

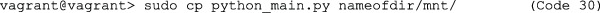

I. You should now be able to run a Singularity container using the directory as your container (Code 31). Note, a sandbox container has no defined %runscript, and thus “singularity exec” is preferred instead of “singularity run”. With “singularity exec” we can run a program from within the container; in our case, we run the file python_main.py with python3.

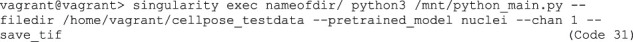

J. If you are satisfied with the functionality of your container and want to build a .sif file for distribution, you can easily do so with a sandbox container (Code 32).

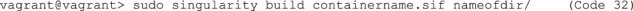

K. You can now use “singularity exec” on the built .sif file to run Cellpose (Code 33).


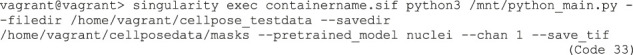


*Note 1:* Sandbox containers have their advantage to iteratively build and test, but definition files are preferred overall to document and share a container.

*Note 2*: To use a different Python version than the default Python version installed with your Ubuntu version (e.g., Python3.6.9 for Ubuntu18.04, or Python3.8 for Ubuntu20.04), first install “software-properties-common” (apt install software-properties-common) to help manage the repositories, then PPA “deadsnakes” which contains different Python versions packaged for Ubuntu (add-apt-repository ppa:deadsnakes/ppa). Next, install the preferred Python version, e.g., “apt install python3.8”. You can test the successful installation by typing “python3.8 --version”. Also, updating pip is important, e.g., “python3.8 -m pip install --upgrade pip”. As there are now two Python versions in the container (one for the operating system and one for the application), consider setting up virtual environments (https://docs.python.org/3/tutorial/venv.html).

*Note 3*: Specific package versions can be installed by specifying the version after the package name. The package version required is often indicated by the code you want to run. Different versions and their release history can be found on the Python Package Index (PyPI) website. For example, if you want to install numpy version 1.20, find it in the release history of PyPI (https://pypi.org/project/numpy/1.20.0/), and write: “pip install numpy==1.20”.

## Discussion

In this paper, we have introduced containers as a powerful tool to make image processing and analysis more reproducible and easily used by users in microscopy labs and imaging facilities with little specific background in scientific computation. As containers are currently hardly used in the imaging field, we have provided a step-by-step protocol for establishing a Singularity container with an exemplary workflow using a state-of-the art segmentation software, Cellpose (Stringer et al., 2021).

Our container example demonstrates how easy it is to run an advanced workflow without installing dependencies. This is particularly useful to quickly test a workflow on images and could be a great way to test published work on your own dataset. Containers could thus satisfy the increasing demand of journals and peer-reviewers to provide easy access to complex image processing and analysis workflows for reproducibility and validation (Lee and Kitaoka 2018; Jost and Waters 2019), Beyond the here provided examples, the provided Singularity definition file easily allows for extension of the capacity of the container to improve the workflow. Should the user want to implement other Python packages or integrate additional software, they can easily install those in the %post section or in writable directories. For more advanced operations such as signing a container or interactive sessions, several comprehensive tutorials exist such as https://singularity-tutorial.github.io/, https://hpc.nih.gov/apps/singularity.html.

In our step-by-step protocol, we relied on a Vagrant Box to build the container on Windows (step 1–12) and the cluster computer (Supplementary Note S1). The Vagrant Box offers the advantage to provide an operating system with a pre-installed Singularity framework that makes establishing containers straight-forward. This might become even easier in the future with Windows Subsystem for Linux (WSL1 and WSL2). Particularly interesting is the feature that WSL2 offers a full Linux kernel on Windows. This might make the step of installing a Vagrant Box obsolete and might provide opportunities to build and run Singularity containers on a Windows operating system.

In conclusion, we envision that Singularity containers will help to overcome the problem of uncertainty in recreating bioimage analysis results. Eventually, all experimental code may be containerized by the scientists running the experiment so that years after publication of the manuscript readers can verify the results easily and test workflows on their own dataset.

## Data Availability

The datasets presented in this study can be found in online repositories. The names of the repository/repositories and accession number(s) can be found in the article/Supplementary Material.
