# Supplementary material for: Singularity Containers Improve Reproducibility and Ease of Use in Computational Image Analysis Workflows
Source: Front Bioinform. 2022 Jan 27;1:757291. doi: 10.3389/fbinf.2021.757291 (PMC9581025; doi:10.3389/fbinf.2021.757291)
Supplement: Supplementary file 1 [file DataSheet1.PDF]

Supplementary Material

1 Supplementary Note 1 – Using the HPC system to build a container

While it worked well for us to build the container on the local Windows operating system, containers can also be built on a HPC system or in the cloud:

1) On our local HPC cluster at UT Southwestern (BioHPC), web desktop terminals are available, with Vagrant already installed. Please contact your cluster administrators about whether similar tools are available on your HPC cluster or whether Vagrant is installed globally. After ensuring Vagrant is installed, initiate a Vagrant Box containing Singularity in your folder on the cluster by:

   ```
   > vagrant init sylabs/singularity-3.5-ubuntu-bionic64
   ```

   Now, follow the steps (4-12) described in the main text to establish a container. Be aware of likely proxy issues (see Supplementary Note 3).

2) An alternative is to use the Singularity remote builder from Sylabs. For this, set up an account first at https://cloud.sylabs.io/builder. In their web interface, recipe files can be directly uploaded. Alternatively, you can connect from the terminal to the remote builder to build your container in the cloud and get it back onto your local system. To do this, generate an access token on the web site and start an interactive session on the cluster:

   a) To start an interactive session on the cluster, type:

   ```
   >srun --nodes=1 --mem=12384 --ntasks-per-node=1 --time=02:00:00 --pty bash -i
   ```

   If required, modify the SLURM parameters based on the expected build time and memory requirements.

   b) Now, load Singularity into your active session:

   ```
   >module load singularity
   ```

   c) Next, move to your folder with the recipe file

   ```
   >cd /your/folder/singularity
   ```

   d) Now, connect to the remote builder. For this, you need to have your access token ready that is generated on the https://cloud.sylabs.io/builder website.

   ```
   >singularity remote login
   ```

   e) Now, adding the flag "--remote" will allow you to build your container without root privileges, e.g:

```
>singularity build --remote output.sif recipefile.recipe
```

Note: There is currently no support to include files from the %files header for remote building of containers. You might however consider building a container that calls a file on the local file system during execution of the container.

2 Supplementary Note 2 – The Vagrant File

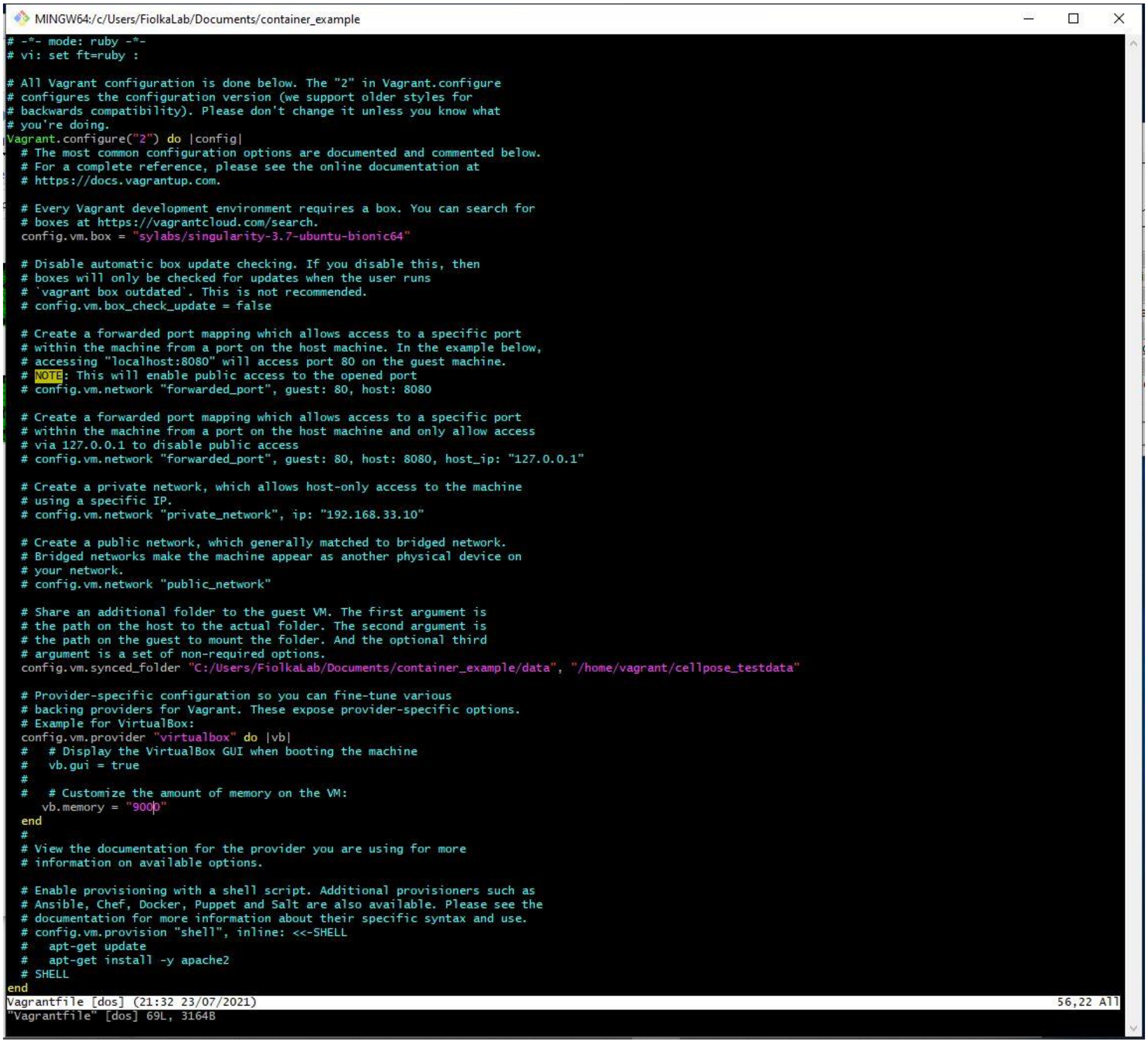

```
MINGW64:/c/Users/FiolkaLab/Documents/container_example
# -*- mode: ruby -*-
# vi: set ft=ruby :

# All Vagrant configuration is done below. The "2" in Vagrant.configure
# configures the configuration version (we support older styles for
# backwards compatibility). Please don't change it unless you know what
# you're doing.
Vagrant.configure("2") do |config|
  # The most common configuration options are documented and commented below.
  # For a complete reference, please see the online documentation at
  # https://docs.vagrantup.com.

  # Every Vagrant development environment requires a box. You can search for
  # boxes at https://vagrantcloud.com/search.
  config.vm.box = "sylabs/singularity-3.7-ubuntu-bionic64"

  # Disable automatic box update checking. If you disable this, then
  # boxes will only be checked for updates when the user runs
  # `vagrant box outdated`. This is not recommended.
  # config.vm.box_check_update = false

  # Create a forwarded port mapping which allows access to a specific port
  # within the machine from a port on the host machine. In the example below,
  # accessing "localhost:8080" will access port 80 on the guest machine.
  # NOTE: This will enable public access to the opened port
  # config.vm.network "forwarded_port", guest: 80, host: 8080

  # Create a forwarded port mapping which allows access to a specific port
  # within the machine from a port on the host machine and only allow access
  # via 127.0.0.1 to disable public access
  # config.vm.network "forwarded_port", guest: 80, host: 8080, host_ip: "127.0.0.1"

  # Create a private network, which allows host-only access to the machine
  # using a specific IP.
  # config.vm.network "private_network", ip: "192.168.33.10"

  # Create a public network, which generally matched to bridged network.
  # Bridged networks make the machine appear as another physical device on
  # your network.
  # config.vm.network "public_network"

  # Share an additional folder to the guest VM. The first argument is
  # the path on the host to the actual folder. The second argument is
  # the path on the guest to mount the folder. And the optional third
  # argument is a set of non-required options.
  config.vm.synced_folder "C:/Users/FiolkaLab/Documents/container_example/data", "/home/vagrant/cellpose_testdata"

  # Provider-specific configuration so you can fine-tune various
  # backing providers for Vagrant. These expose provider-specific options.
  # Example for VirtualBox:
  config.vm.provider "virtualbox" do |vb|
  #   # Display the VirtualBox GUI when booting the machine
  #   vb.gui = true
  #
  #   # Customize the amount of memory on the VM:
     vb.memory = "9000"
  end
  #
  # View the documentation for the provider you are using for more
  # information on available options.

  # Enable provisioning with a shell script. Additional provisioners such as
  # Ansible, Chef, Docker, Puppet and Salt are also available. Please see the
  # documentation for more information about their specific syntax and use.
  # config.vm.provision "shell", inline: <<-SHELL
  #   apt-get update
  #   apt-get install -y apache2
  # SHELL
end
Vagrantfile [dos] (21:32 23/07/2021)                                56,22 All
"Vagrantfile" [dos] 69L, 3164B
```

Supplementary Figure 1. A screenshot of the Vagrantfile for running the code

3 Supplementary Note 3 – Setting up a Singularity Container behind a firewall

Note: If you are behind a firewall, make sure that the proxy settings in Windows and for Git / Git Bash are properly set.

A. In case your computer is behind a firewall, the “vagrant up” command might lead to an error message. In this case, proceed as follows:

1) Download the sylabs/singularity-3.7-ubuntu-bionic64 box from the website https://app.vagrantup.com/sylabs/boxes/singularity-3.7-ubuntu-bionic64 by clicking on the blue download arrow

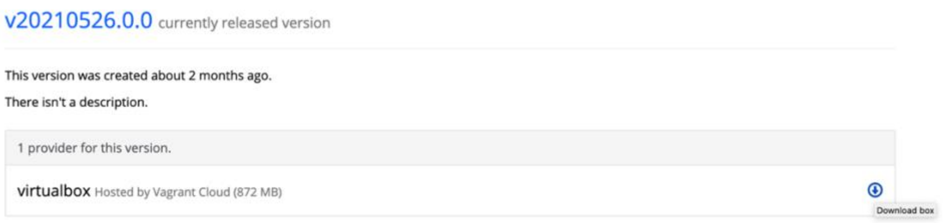

2) Now, add the downloaded box:

```
> vagrant box add -name="sylabs/singularity-3.7-ubuntu-bionic64"
X://path/to/downloadedfile/7fe1832a-71a4-41d1-bd73-8f98759bee58
```

3) Now you can use vagrant up to run the Vagrant Box that contains Singularity.

B. Moreover, when building the container inside the Vagrant box with “sudo singularity build” proxy issues can arise.

1) Exit the Vagrant Box by typing “exit”

2) Install the plugin vagrant-proxyconf

```
> vagrant plugin install vagrant-proxyconf
```

3) Modify the Vagrantfile with vim by inserting at the beginning after “Vagrant.configure("2") do |config|“:

```
  if Vagrant.has_plugin?("vagrant-proxyconf")
    config.proxy.http     = "http://[your-proxy-address.edu]:[portnumber]/"
    config.proxy.https    = "http://[your-proxy-address.edu]:[portnumber]/"
    config.proxy.no_proxy = "localhost,127.0.0.1,.example.com"
  end
  # ... rest of the configurations
```

4) Next, update vagrant with:

```
> vagrant up
```

5) Now, connect to the Vagrant Box again with ”vagrant ssh”
