# Supplementary figures and images for: Singularity Containers Improve Reproducibility and Ease of Use in Computational Image Analysis Workflows
Source: Front Bioinform. 2022 Jan 27;1:757291. doi: 10.3389/fbinf.2021.757291 (PMC9581025; doi:10.3389/fbinf.2021.757291)

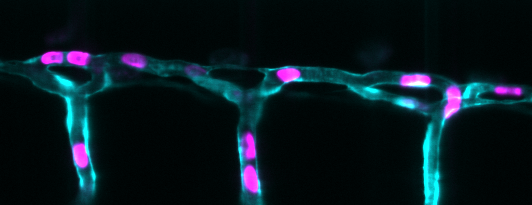

Supplement: Supplementary file 2 [file DataSheet2.ZIP › Containerize_ImageAnalysis-main/data/vessel_bloodflow1.tif]

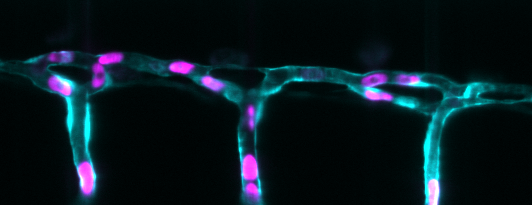

Supplement: Supplementary file 2 [file DataSheet2.ZIP › Containerize_ImageAnalysis-main/data/vessel_bloodflow2.tif]
